# The effect of traditional malting technology practiced by an ethnic community in northern Uganda on in‐vitro nutrient bioavailability and consumer sensory preference for locally formulated complementary food formulae

**DOI:** 10.1002/fsn3.856

**Published:** 2018-10-18

**Authors:** Docus Alowo, Christopher Muggaga, Duncan Ongeng

**Affiliations:** ^1^ Department of Food Science and Postharvest Technology Faculty of Agriculture and Environment Gulu University Gulu Uganda

**Keywords:** anti‐nutritional factors, complementary food, nutrient bioavailability, sensory preference, traditional malting technology

## Abstract

The occurrence of anti‐nutritional constituents in plants is an important factor that negatively affects bioavailability of nutrients and effectiveness of plant‐based foods in complementary feeding in rural areas in developing countries. However, proven methods that improve bioavailability of nutrients and tailored for application in processing complementary foods among rural communities are largely lacking. This study examined the efficacy of a traditional malting technology practiced by the Acholi ethnic community of northern Uganda to improve protein digestibility and bioavailability of iron and zinc from millet–sesame–soy composite containing 200, 300, and 550 kcal meant for complementary feeding of children aged 6–8, 9–12, and 13–23 years old, respectively. The technology involves washing and soaking of ingredients for 12 hr; malting ingredients individually for 48 hr with water changed after every 6 hr; and sun‐drying malted ingredients for 72 hr. Results showed that the level of anti‐nutritional factors significantly reduced (*p* ≤ 0.05) in all the composite formulae except the content of total phenolics in 200, tannins in 300 and 550 kcal, composite formula, respectively. In vitro protein digestibility significantly improved (*p* ≤ 0.05) in all the composite formulae except in the 200 kcal formula. Iron bioavailability significantly increased (*p* ≤ 0.05) in all the composite formulae except in the 550 kcal energy category. Improvement in zinc bioavailability was only observed in the 300 kcal formula. However, there were significant reductions (*p* ≤ 0.05) in the level of caregiver preferences for sensory properties and overall acceptability of the composites. These results demonstrate that the traditional malting technology has potential to improve nutrient bioavailability in plant‐based foods but requires improvement in order to increase its efficacy and mitigate negative effects on sensory appeal.

## 1 INTRODUCTION

Protein‐energy malnutrition and micronutrient deficiencies have remained a significant public health problem among children in Sub‐Saharan Africa (Andersson, Karumbunathan, & Zimmermann, 2012). Recent statistics show that the level of child undernutrition in the sub‐continent is one of the highest globally. Specifically, the level of stunting, wasting, and underweight has been found in the range of 37.9%–57.7%, 10.5%–15.5%, and 25.0%–36.4%, respectively (Akombi, Agho, Merom, Renzaho, & Hall, 2017). Generally, the prevalence of child undernutrition and associated health problems in low‐income countries such as those in Sub‐Saharan Africa is known to peak at 6 months of age when complementary foods are introduced in the child's diet (Saha et al., 2008). This is mainly due to factors such as poor feeding practices, poor food preparation and handling practices, low energy density in infant formulae and poor dietary diversity (Fasuan, Fawale, Enwerem, Uche, & Ayodele, 2017; Turyashemererwa, Kikafunda, & Agaba, 2009).

Nonetheless, complementary feeding is essential because at the age of 6 months, the child's nutrient and energy demands outweigh what can be supplied by breastmilk (Agostoni et al., 2008). Therefore, adequate and appropriately administered complementary foods should ensure proper growth and development of children. A number of standardized complementary food formulae are commercially available (Dewey & Adu‐afarwuah, 2008). In Sub‐Saharan Africa and other low‐income regions of the world, standardized commercial complementary food formulae are too expensive and can only be accessed by economically advantaged households that are mostly located in urban areas (Tizazu, Urga, Abuye, & Retta, 2010). However, poor households that constitute the majority in the sub‐continent and other low‐income regions of the world are concentrated in rural areas and cannot access such expensive infant formulae (Anoma, Collins, & McNeil, 2014).

The use of locally available food resources of both plant and animal origin has been recommended as a key strategy for improving complementary feeding among poor households in low‐income countries such as those located in Sub‐Saharan Africa (Kounnavong et al., 2011). Such food resources can be compounded in appropriate proportions to make nutritious composite formulae. However, it is important to recognize that in rural areas in Sub‐Saharan Africa, food resources available are mainly plant‐based (Hotz & Gibson, 2001). Therefore, complementary foods made locally in rural settings are largely of plant origin. One of the key constrains associated with plant‐based foods is the existence of anti‐nutritional factors (e.g., phytates, trypsin inhibitors, tannins, oxalates, and total phenolic compounds) that limit nutrient bioavailability and utilization (Krishnan, Dharmaraj, & Malleshi, 2012; Mugendi, Njagi, Kuria, Mwasaru, & Mureithi, 2010). Protein is the main macronutrient that is highly affected by trypsin inhibitors in legumes (McEwan, Shangase, Djarova, & Opoku, 2014) while iron and zinc are micronutrients of public health importance whose bio‐availabilities are significantly reduced by plant anti‐nutritional constituents such as phytates, tannins, polyphenols, and oxalates (Gemede & Ratta, 2014; Mamiro, Mwanri, Mamiro, Nyagaya, & Ntwenya, 2016).

Several strategies such as soaking, malting, fermentation, and roasting have been proposed for improving nutrient bioavailability in plant‐based foods at household level. Malting is an important strategy which has been shown to improve bioavailability of both macro‐ and micro‐nutrients in plant‐based foods (Cornwell, Cohick, & Raskin, 2004; Mugendi et al., 2010). Improvement in bioavailability effected by malting is mainly due to reduction in the levels of anti‐nutritional factors (Ikujenlola, 2014; James, Oloyede, Ocheme, Chinma, & Agbejule, 2015; Nkundabombi, Nakimbugwe, & Muyonga, 2015; Onyango et al., 2013; Thapliyal & Singh, 2015). Nevertheless, it should be noted that published information on malting has mainly been derived from experiments conducted under controlled conditions in the laboratory. Such results may be difficult to adapt for practical application in rural settings because laboratory‐controlled conditions cannot easily be replicated in rural environment where conditions are generally very dynamic.

In many countries in Sub‐Saharan Africa, rural communities apply traditional malting treatment in food processing (Fasuan et al., 2017; Fikiru, Bultosa, Forsido, & Temesgen, 2016). Traditional malting practices differ between countries and also vary among communities within a country (Bokulich & Bamforth, 2013; Krishnan et al., 2012). A common observation among rural communities is that malting is largely applied in processing alcoholic beverages and foods meant for general consumption. However, inadequate information exists on the application of traditional malting technologies practiced in rural areas in processing complementary food formulae. Fundamentally, processing is known to alter sensory properties of food and consumer preference in turn (Fikiru et al., 2016) and these changes depend on the processing method applied (Ariahu, Ingbian, & Ojo, 2009; Laurie & Van Heerden, 2012). To provide an insight into the potential of traditional malting technologies in processing plant‐based complementary foods in rural areas, this study examined the effect of a traditional malting technology practiced in Acholi Sub‐region of Uganda on: (a) in vitro protein digestibility and bioavailability of iron and zinc; and (b) consumer sensory preference for millet–sesame–soy composite formulae.

## 2 MATERIALS AND METHODS

Millet (*Eleusine coracana*), sesame (*Sesanum indicum*) and soybean (*Glycine max*) used in the study were purchased from local markets. Malting technology practiced in Acholi sub‐region has not been documented before. Therefore, focus group discussions (FGDs) were held to generate the information. Eight FGDs were conducted, one in each of the eight districts (Gulu, Amuru, Nwoya, Kitgum, Lamwo, Agago, Pader, and Omoro) that comprise Acholi Sub‐region. This number (8) of FGDs is above the minimum number (6) required for saturation of information in qualitative studies (Tong, Sainsbury, & Craig, 2007). Each FGD had 10 participants and lasted for 60 min. The participants consisted of caregivers of children aged 6–23 months and were purposively selected. The outcome of the FGD revealed that traditional malting technology practiced by the Acholi ethnic community involves the following: (a) washing and soaking of ingredients for 12 hr; (b) malting ingredients individually for 48 hr (with water changed after every 6 hr); and (c) sun‐drying malted ingredients for 72 hr.

An experiment was then set‐up in the community with two treatments: (a) complementary food formulae ingredients subjected to the malting process described above; and (b) control, not subjected to the malting process. Malting was conducted separately for each ingredient following the procedure documented during the FGD. Thereafter, each ingredient was roasted separately making use of indigenous knowledge of the study participants. Roasting took 15, 25, and 30 min for millet, sesame, and soy, respectively. Roasted ingredients were blended to yield flour composite formulae matching the recommended daily allowance (RDA) for energy level of 200, 300, and 550 kcal meant for children aged 6–8, 9–11, and 12–23 months, respectively as recommended by WHO (Dewey, 2001). Calculation of the required weight of each ingredient to constitute the composite formula for each energy category was performed using Harvest Plus Food Composition Table (Hotz, Lubowa, Sison, Moursi, & Loechl, 2012) and Microsoft Excel software version 2010. The mixing proportions of the different formulations for each energy category were thus: (a) 76% millet–3.8% sesame–20.2% soybean for 200 kcal; (b) 76.3% millet–3.2% sesame–20.5% soybean for 300 kcal; and (c) 77% millet–3% sesame–20% soybean for 550 kcal composite (Table 1). These mixing proportions were used on the basis of a preliminary study which showed that they yielded products that were most preferred and accepted by caregivers in the study area. These mixing propositions are also in line with results of other studies which showed that composite flours with higher proportion of staple cereals were more preferred (Laminu, Modu, & Numan, 2011; Olagunju & Ifesan, 2013).

**Table 1:** Ingredient composition for millet–sesame–soy composite flour corresponding to the recommended daily energy requirements for children aged 6–8, 9–11, and 12–24 months

Ingredients used for composites	Reference energy (kcal)/100 g for roasted ingredients	Weight/g	Energy/kcal	% kcal ratios	% Gram ratios
**E11**	**200 kcal**				
Millet	354	39.5	139.8	70	76.0
Sesame	565	2	11.3	5	3.8
Soybean	474	10.5	49.5	25	20.2
Total		52^a^	200.6^b^	100	100
**E21**	**300 kcal**				
Millet	354	59.5	210.6	70	76.3
Sesame	565	2.5	14.1	5	3.2
Soybean	474	16	75.4	25	20.5
Total		78^a^	300.1^b^	100	100
**E31**	**550 kcal**				
Millet	354	109	385.9	70	76.2
Sesame	565	5	28.3	5	3.5
Soybean	474	29	136.6	25	20.3
Total		143^a^	550.7^b^	100	100.0

Notes

E1, E21, and E31 are composite formulae corresponding with energy level of 200, 300, and 550 kcal meant for children aged 6–8, 9–11, and 12–23 months, respectively.

^a^Amount in grams of the composite flour expected to meet the recommended energy requirement for children in each age category. ^b^Calculated energy for each composite formula for each age category based on Harvest Plus Food Composition Table (Hotz et al., 2012).

Compounded ingredients for each formula were ground to 0.25 mm particle size using a 20HP mill (AMEC mill, Beijing, China). The mill was thoroughly cleaned before and after each sample preparation in order to avoid cross‐mixing of samples between different treatments. Following preparation, the samples were subjected to: (a) laboratory analysis to determine the contents of total tannins, phytates, polyphenols, trypsin inhibitor activity, and in vitro protein digestibility and bioavailability of iron and zinc; and (b) sensory evaluation to assess consumer sensory preference for aroma, color, taste, mouthfeel, thickness, and overall acceptability. The content of total tannins, phytates, polyphenols was analyzed according to the method previously reported by Polshettiwar, Ganjiwale, Wadher, and Yeole (2007), Onweluzo and Nwabugwu (2009), and Beta, Nam, Dexter, and Sapirstein (2005), respectively. Trypsin inhibitor activity and in vitro protein digestibility were assessed according to Van Eys (2005) and Alka, Neelam, and Shruti (2012), respectively. In vitro bioavailability of iron and zinc was determined according to the enzymatic method previously described by Ting and Loh (2016). The experiment was replicated three times, and each analysis was performed in duplicates.

Sensory evaluation was conducted using a 5‐point hedonic scale (from 1—dislike very much, 2—dislike moderately, 3—neither like nor dislike, 4—like moderately, 5—like very much) as previously described by Lim (2011). Participants involved consisted of caregivers of children 6–23 months who did not participate in processing the formulae. However, those with children below 6 or above 23 months of age were excluded from the study. Caregivers were considered as main respondents because of their ability to objectively evaluate the sensory characteristics of the formulations (Amankwah, Barimah, Nuamah, Oldham, & Nnaji, 2009) compared to children in the age category of 6–23 months. A total of 50 caregivers participated in the evaluation exercise and are consistent with the number used in a previous study (Amankwah et al., 2009). Sensory evaluation was conducted using porridge samples prepared from the composite flour formulae. All porridge samples were made to the recommended spoonable consistency (WHO, 2009) corresponding to an apparent viscosity of 1–3 Pa.s (Amagloh et al., 2013; Dewey, 2001; Mouquet & Trèche, 2001). To achieve this, each composite flour was mixed with clean water at 40% and 20% w/v for malted and un‐malted products, respectively, and cooked for 15 min. Before actual evaluation exercise, recruited caregivers were oriented on the definition of the attributes tested. Evaluation samples were then served in separate disposable cups. Each participant was served with clean warm water to rinse his/her mouth before and after tasting each porridge sample to prevent over/under scaling of the sensory characteristics (Tizazu et al., 2010). Participants were positioned far away from each other to ensure individual scaling without external interference. Finally, participants were asked to choose one best formula out of the two formulae (control, malted) served for each energy category according to their degree of liking.

### 2.1 Statistical analysis

Paired sample *t* test was used to compare the levels of anti‐nutritional factors, in vitro protein digestibility, in vitro bioavailability of iron and zinc, and the level of rating of each sensory parameter between the malted and unmated (control) products for each energy category. The level of statistical significance was fixed at 5% (*p* ≤ 0.05), and analysis was performed using SPSS version 19.

## 3 RESULTS

Table 2 presents data on the contents of anti‐nutritional factors in malted and un‐malted composite formulae for different energy categories. The effect of malting on the content of anti‐nutritional factors was dependent on the composite formula type (energy category) and specific anti‐nutritional factor in question. For composite formula of 200 kcal, the content of phytates, tannins, and level of trypsin inhibitor activity in malted formula was significantly lower than observed in un‐malted formula by about 0.42%, 0.5%, 2.76%, respectively. In the case of 300 kcal composite formula, the content of total polyphenols, phytates, and level of trypsin inhibitor activity observed in malted composite was significantly lower (*p *≤* *0.05) than detected in un‐malted composite by 0.24%, 0.92%, and 1.7%, respectively. Malting did not significantly (*p *>* *0.05) affect the polyphenolic and tannin content of 200 and 300 kcal composites, respectively. For 550 kcal composite formula, the content of total polyphenols and phytates in malted composite was significantly lower (*p* ≤ 0.05) than observed in un‐malted samples by about 0.17% and 1.2%, respectively. Nevertheless, malting had no significant effect (*p* > 0.05) on tannin content and the level of trypsin inhibitor activity in the 550 kcal composite formula.

**Table 2:** Content of anti‐nutritional factors in malted and un‐malted millet–sesame–soy composite formulae segregated by energy category

Parameter	Treatment					
	E11 (200 kcal)		E21 (300 kcal)		E31 (550 kcal)	
	E11M	E11C	E21M	E21C	E31M	E31C
Total phenolic (%)	0.28 ± 0.00^a^	0.31 ± 0.00^a^	0.02 ± 0.01^a^	0.26 ± 0.00^b^	0.00 ± 0 .00^a^	0.17 ± 0.00^b^
Phytates (%)	3.65 ± 0.16^a^	4.07 ± 0.16^b^	1.48 ± 0.14^a^	2.40 ± 0.08^b^	0.88 ± 0.08^a^	2.03 ± 0.14^b^
Tannins (%)	0.88 ± 0.25^a^	1.38 ± 0.08^b^	0.72 ± 0.25^a^	0.97 ± 0.17^a^	0.88 ± 0.08^a^	0.97 ± 0.00^a^
Trypsin inhibitor (%)	0.00 ± 0.00^a^	2.76 ± 0.29^b^	0.00 ± 0.00^a^	1.67 ± 0.29^b^	0.79 ± 0.18^a^	0.93 ± 0.12^a^

Note

Values show mean ± *SD* (*n* = 6). For each energy category and for each parameter, means in the same row followed by different superscripts are significantly different at 5% level of significance (*p* ≤ 0.05). E11M: composite formulae for the 200 kcal energy category (76% millet–3.8% sesame–20.2% soybean) subjected to malting; E11C: composite formulae for the 200 kcal energy category (76% millet–3.8% sesame–20.2% soybean) used as control for E11M; E21M: composite formulae for the 300 kcal energy category (76.3% millet–3.2% sesame–20.5% soybean) subjected to malting; E21C: composite formulae for the 300 kcal energy category (76.3% millet–3.2% sesame–20.5% soybean) used as control for E21M; E31M: composite formulae for the 550 kcal energy category (77% millet–3% sesame–20% soybean) subjected to malting; E31C: composite formulae for the 550 kcal energy category (77% millet–3% sesame–20% soybean) used as control for E31M.

The results of in vitro protein digestibility and bioavailability of iron and zinc are presented in Table 3. The proportion of digestible protein in 300 and 550 kcal composite significantly increased (*p* ≤ 0.05) by 15.9 and 52.3 units, respectively after malting, but no effect (*p* > 0.05) was observed on the 200 kcal composite formula. The proportion of bioavailable zinc in malted samples for 300 kcal composite was significantly above (*p* ≤ 0.05) what was recorded from un‐malted samples by about 9.5 units, while no difference (*p* > 0.05) was observed for both the 200 and 550 kcal composites. With regard to iron bioavailability, the proportion of bioavailable mineral significantly increased (*p* ≤ 0.05) in all the composite formulae except in the 550 kcal formulae following malting. The increase was highest in 300 kcal composite (19.4 units) followed by 200 kcal composite (17.6 units).

**Table 3:** In vitro protein digestibility and bioavailability of iron and zinc from millet–sesame–soy composite formulae segregated by energy category

Parameter	Treatment					
	E11 (200 kcal)		E21(300 kcal)		E31(550 kcal)	
	E11M	E11C	E21M	E21C	E31M	E31C
% digestible protein	43.47 ± 5.24^a^	42.86 ± 1.32^a^	58.76 ± 14.00^a^	42.90 ± 9.89^b^	73.21 ± 0.00^a^	20.93 ± 0.01^b^
% bioavailable zinc	14.22 ± 2.80^a^	14.23 ± 2.84^a^	19.75 ± 4.09^a^	10.25 ± 1.68^b^	17.83 ± 6.54^a^	13.12 ± 0.94^a^
% bioavailable iron	21.26 ± 7.92^a^	3.68 ± 1.93^b^	21.14 ± 2.26^a^	1.70 ± 0.12^b^	22.66 ± 11.36^a^	9.97 ± 3.88^a^

Note

Values show mean ± *SD* (*n* = 6). For each energy category and for each parameter, means in the same row followed by different superscripts are significantly different at 5% level of significance (*p* ≤ 0.05). E11M: composite formulae for the 200 kcal energy category (76% millet–3.8% sesame–20.2% soybean) subjected to malting; E11C: composite formulae for the 200 kcal energy category (76% millet–3.8% sesame–20.2% soybean) used as control for E11M; E21M: composite formulae for the 300 kcal energy category (76.3% millet–3.2% sesame–20.5% soybean) subjected to malting; E21C: composite formulae for the 300 kcal energy category (76.3% millet–3.2% sesame–20.5% soybean) used as control for E21M; E31M: composite formulae for the 550 kcal energy category (77% millet–3% sesame–20% soybean) subjected to malting; E31C: composite formulae for the 550 kcal energy category (77% millet–3% sesame–20% soybean) used as control for E31M.

The results on the effect of malting on caregiver preference for sensory attributes and overall acceptability of the composite formulae are presented in Table 4. In general, the scores for sensory attributes of all the malted composites were significantly lower (*p* ≤ 0.05) than for un‐malted composites irrespective of the energy category. The degree of liking for sensory attributes of the composites was at least 60% and 80% for malted and un‐malted products, respectively and was similar across energy category.

**Table 4:** Caregivers’ scores for sensory attributes and overall acceptability of malted and un‐malted millet–sesame–soy composite segregated by energy category

Sensory attribute	Treatment					
	E11 (200 kcal)		E21 (300 kcal)		(E31) 550 kcal	
	E11M	E11C	E21M	E21C	E31M	E31C
Color	3.89 ± 1.36^a^	4.75 ± 0.46^b^	4.20 ± 1.18^a^	4.50 ± 0.95^b^	4.35 ± 1.02^a^	4.75 ± 0.70^b^
Aroma	3.61 ± 1.29^a^	4.69 ± 0.69^b^	4.08 ± 1.17^a^	4.58 ± 0.91^b^	3.98 ± 1.38^a^	4.86 ± 0.53^b^
Thickness	3.59 ± 1.38^a^	4.42 ± 0.89^b^	4.03 ± 1.14^a^	4.47 ± 1.02^b^	3.87 ± 1.33^a^	4.70 ± 0.67^b^
Mouthfeel	3.25 ± 1.23^a^	4.55 ± 0.76^b^	4.07 ± 1.06^a^	4.18 ± 1.13^a^	3.62 ± 1.41^a^	4.56 ± 1.02^b^
Taste	3.25 ± 1.35^a^	4.54 ± 0.82^b^	3.86 ± 1.19^a^	4.43 ± 1.00^b^	3.49 ± 1.40^a^	4.68 ± 0.82^b^
Overall acceptability	3.35 ± 1.43^a^	4.63 ± 0.65^b^	3.92 ± 1.08^a^	4.44 ± 0.97^b^	3.55 ± 1.37^a^	4.57 ± 0.77^b^

Note

Values show mean ± *SD* (*n* = 6). For each energy category and each sensory attribute, means in the same row followed by different superscripts are significantly different at 5% level of significance (*p* ≤ 0.05). E11M: composite formulae for the 200 kcal energy category (76% millet–3.8% sesame–20.2% soybean) subjected to malting; E11C: composite formulae for the 200 kcal energy category (76% millet–3.8% sesame–20.2% soybean) used as control for E11M; E21M: composite formulae for the 300 kcal energy category (76.3% millet–3.2% sesame–20.5% soybean) subjected to malting; E21C: composite formulae for the 300 kcal energy category (76.3% millet–3.2% sesame–20.5% soybean) used as control for E21M; E31M: composite formulae for the 550 kcal energy category (77% millet–3% sesame–20% soybean) subjected to malting; E31C: composite formulae for the 550 kcal energy category (77% millet–3% sesame–20% soybean) used as control for E31M.

## 4 DISCUSSION

Whereas the need for rural communities in developing countries to use locally available food resources to produce complementary foods has been widely recognized (Hotz & Gibson, 2007), limited attention has been given to development of user‐friendly processing methods that can improve bioavailability of nutrients from plant‐based foods. This is notwithstanding the fact that due to poor socio‐economic conditions in rural areas in developing countries, complementary foods in such localities are largely plant‐based (Tizazu et al., 2010). To provide an insight into what can be practically possible in rural areas, this study evaluated the potential of a traditional malting technology practised in Acholi Sub‐region of Uganda to improve bioavailability of protein, iron, and zinc from a typical plant‐based complementary food formulae.

Fundamentally, improvement in nutrient bioavailability from plant‐based foods is a function of the extent to which a processing method inactivates or reduces the content of specific anti‐nutritional factors (Chaudhary & Vyas, 2014; Gautam, Platel, & Srinivasan, 2010; Mamiro et al., 2016). Generally, there was a decrease in the level of anti‐nutritional factors following application of the traditional malting technology despite a few exceptional situations of non‐effects. Nonetheless, a critical look at the results indicates that efficacy of the traditional malting technology on the content of certain anti‐nutritional factors was comparable to results of other studies based on experiments conducted under controlled conditions in the laboratory. For instance, percentage reduction in phytate content of the millet‐sesame‐soy composite formulae (1.2%–9.2%) recorded in the current study is higher than reduction level (1.1%–3.1%) achieved by Hemalatha, Platel, and Srinivasan (2007) after malting cereals and legumes for 48 hr at 25°C. A similar observation is also true for tannin content. In this case, the extent of reduction in the level of tannin recorded in the current study (9%–50%) is also better than what had been reported (7%–14%) for legumes based on laboratory‐controlled experiments conducted by Ghavidel and Prakash (2007). These scenarios clearly illustrate the potential of traditional rural‐based malting practices for improving bioavailability of nutrients from plant‐based complementary foods, and indeed the need to consider research on application of such technologies in processing plant‐based complementary foods in rural areas in developing countries. The inability of the traditional technology to reduce the levels of some of the anti‐nutritional factors in certain composite formulae indicates the limitation of the technology but at the same time provides opportunity for research to improve its efficacy.

Despite the limited efficacy of the traditional malting technology in reducing the level of anti‐nutritional factors in all the composite formulae investigated, the technology was capable of improving in vitro bioavailability of nutrients investigated except in certain cases. Similar results were also reported by various authors who dealt with different plant‐based food products (Anigo, Ameh, Ibrahim, & Danbauchi, 2010; Nkundabombi et al., 2015; Onyango et al., 2013). More interestingly, the level of improvement in iron and zinc bioavailability observed in the current study is much higher (12.69–19.44 and 0.01–9.5 units, respectively) compared to what has been reported before when legumes and cereals were malted under controlled conditions in the laboratory (3.13%–4.9% increase in iron bioavailability and 1.5%–16.46% decrease in zinc bioavailability) (Hemalatha et al., 2007). This further illustrates the potential of the traditional technology and justifies the need for further studies to improve its efficacy. Nonetheless, it is interesting to note that, whereas the ingredients used to produce the composite formulae were subjected to the same malting treatment, the effect on nutrient bioavailability was heterogeneous and dependent on the composite formula type. This observation could be attributed to low degree of reproducibility of the traditional technology because the process is largely uncontrolled. This is reflected in the high standard deviations recorded for some of the results. Future studies should consider improving the reproducibility of the technology.

It is generally known that processing methods applied in the preparation of complementary food composite blends greatly influence sensory quality and overall acceptability of the final product (Makanjuola, Ogunmodede, Makanjuola, & Awonorin, 2012). Results of the current study indicate significantly lower scores for sensory attributes of malted samples compared to the un‐malted samples. The observed negative effect of malting on consumer sensory appeal is not peculiar to this study. It has also been reported in other studies conducted under controlled conditions in the laboratory (Gernah, Ariahu, & Ingbian, 2012; Nkundabombi et al., 2015). Unfortunately, limited attention has been given to improving sensorial quality of malted complementary foods. Considering the fact that acceptability of food is largely driven by sensory appeal compared to nutritional quality (Laurie & Van Heerden, 2012), the observed negative sensorial effect is a potential factor that should be critically considered in future attempts to improve the efficacy of the traditional malting technology for enhancing bioavailability of nutrients from plant‐based foods. A major limitation of this study is that bioavailability of iron and zinc was assessed after malting and roasting treatments. Therefore, it still remains questionable as to whether the cooking treatment applied to transform the composite flours to porridges did not exert additional effect on the bioavailability of the nutrients studied.

## 5 CONCLUSIONS

This study has demonstrated that traditional malting technology practiced in Acholi sub‐region of Uganda has potential to improve nutrient bioavailability from plant‐based complementary foods. However, improvements in nutrient bioavailability are associated with reduced product sensorial appeal. Future studies should consider improving the efficacy of the technology on nutrient bioavailability concomitant with improvement in product sensorial appeal.

## CONFLICT OF INTEREST

The authors declare no conflict of interest.

## ETHICAL REVIEW

This study was approved by Gulu University Research Ethics Committee (GUREC 03/03/2017).

## References

[fsn3856-bib-0001] Agostoni, Ã. C. , Decsi, T. , Fewtrell, M. , Goulet, O. , Kolacek, S. , Koletzko, B. , & Van Goudoever, J. (2008). Complementary feeding: A commentary by the ESPGHAN committee on nutrition. Journal of Pediatric Gastroenterology and Nutrition, 46(1), 99–110. 10.1097/01.mpg.0000304464.60788.bd 18162844

[fsn3856-bib-0002] Akombi, B. J. , Agho, K. E. , Merom, D. , Renzaho, A. M. , & Hall, J. (2017). Child malnutrition in sub‐Saharan Africa: A meta‐analysis of demographic and health surveys (2006–2016). PLoS ONE, 12(5), e0177338 10.1371/journal pone.0177338 10.1371/journal 28494007 PMC5426674

[fsn3856-bib-0003] Alka, S. , Neelam, Y. , & Shruti, S. (2012). Effect of fermentation on physicochemical properties & in vitro starch and protein digestibility of selected cereals. International Journal of Agricultural and Food Science, 2(3), 66–70.

[fsn3856-bib-0004] Amagloh, F. K. , Mutukumira, A. N. , Brough, L. , Weber, J. L. , Hardacre, A. , & Coad, J. (2013). Carbohydrate composition, viscosity, solubility, and sensory acceptance of sweetpotato‐ and maize‐based complementary foods. Food & Nutrition Research, 1(57), 18717 10.3402/fnr.v57i0.18717 10.3402/fnr.v57i0.18717 PMC3600427 23516115

[fsn3856-bib-0005] Amankwah, E. A. , Barimah, J. , Nuamah, A. K. M. , Oldham, J. H. , & Nnaji, C. O. (2009). Formulation of weaning food from fermented maize, rice, soybean and fishmeal. Pakistan Journal of Nutrition, 8(11), 1747–1752. 10.3923/pjn.2009.1747.1752

[fsn3856-bib-0006] Andersson, M. , Karumbunathan, V. , & Zimmermann, M. B. (2012). Global Iodine status in 2011 and trends over the past decade. The Journal of Nutrition, 142(4), 744–750. 10.3945/jn.111.149393 22378324

[fsn3856-bib-0007] Anigo, K. M. , Ameh, D. A. , Ibrahim, S. , & Danbauchi, S. S. (2010). Nutrient composition of complementary food gruels formulated from malted cereals, soybeans and groundnut for use in North‐western Nigeria. African Journal of Food Science, 4(3), 65–72.

[fsn3856-bib-0008] Anoma, A. , Collins, R. , & McNeil, D. (2014). The value of enhancing nutrient bioavailabity of lentils: The Sri Lankan scenario. African Journal of Food Agriculture Nutrition and Development, 14(7), 9529–9543.

[fsn3856-bib-0009] Ariahu, C. C. , Ingbian, E. K. , & Ojo, M. (2009). Effects of germination and fermentation on the quality characteristics of maize/mushroom based formulation. African Journal of Food Agriculture Nutrition and Development, 9(5), 1258–1275.

[fsn3856-bib-0010] Beta, T. , Nam, S. , Dexter, J. E. , & Sapirstein, H. D. (2005). Phenolic content and antioxidant activity of pearled wheat and roller‐milled fractions. Cereal Chemistry, 82(4), 390–393. 10.1094/CC-82-0390

[fsn3856-bib-0011] Bokulich, N. A. , & Bamforth, C. W. (2013). The microbiology of malting and brewing. Microbiology and Molecular Biology Reviews, 77(2), 157–172. 10.1128/MMBR.00060-12 23699253 PMC3668669

[fsn3856-bib-0012] Chaudhary, N. , & Vyas, S. (2014). Effect of germination on proximate composition and antinutritional factor of millet (ragi) premixes. International Journal of Food and Nutritional Sciences, 3(4), 72–76.

[fsn3856-bib-0013] Cornwell, T. , Cohick, W. , & Raskin, I. (2004). Dietary phytoestrogens and health. Phytochemistry, 65, 995–1016. 10.1016/j.phytochem.2004.03.005 15110680

[fsn3856-bib-0014] Dewey, K. (2001). Guiding principles for complementary feeding of the breastfed child. Washington, DC: Pan American Health Organization, Division of Health Promotion and Protection, Food and Nutrition Program.

[fsn3856-bib-0015] Dewey, K. G. , & Adu‐afarwuah, S. (2008). Systematic review of the efficacy and effectiveness of complementary feeding interventions in developing countries. Maternal and Child Nutrition, 4, 24–85. 10.1111/j.1740-8709.2007.00124.x 18289157 PMC6860813

[fsn3856-bib-0016] Fasuan, T. O. , Fawale, S. O. , Enwerem, D. E. , Uche, N. , & Ayodele, E. A. (2017). Physicochemical, functional and economic analysis of complementary food from cereal, oilseed and animal polypeptide. International Food Research Journal, 24(1), 275–283.

[fsn3856-bib-0017] Fikiru, O. , Bultosa, G. , Forsido, S. F. , & Temesgen, M. (2016). Nutritional quality and sensory acceptability of complementary food blended from maize (Zea mays), roasted pea (Pisum sativum), and malted barley (Hordium vulgare). Food Science & Nutrition, 5(2), 173–181. 10.1002/fsn3.376 28265352 PMC5332271

[fsn3856-bib-0018] Gautam, S. , Platel, K. , & Srinivasan, K. (2010). Higher bioaccessibility of iron and zinc from food grains in the presence of garlic and onion. Journal of Agriculture And Food Chemistry, 58, 8426–8429. 10.1021/jf100716t 20597543

[fsn3856-bib-0019] Gemede, H. F. , & Ratta, N. (2014). Antinutritional factors in plant foods: Potential health benefits and adverse effects. International Journal of Nutrition and Food Sciences, 3(4), 284–289. 10.11648/j.ijnfs.20140304.18 10.11648/j.ijnfs.20140304.18

[fsn3856-bib-0020] Gernah, D. I. , Ariahu, C. C. , & Ingbian, E. K. (2012). Nutritional and sensory evaluation of food formulations from malted and fermented maize fortified with defatted sesame flour. African Journal of Food Agriculture Nutrition and Development, 12(6), 11–16.

[fsn3856-bib-0021] Ghavidel, R. A. , & Prakash, J. (2007). The impact of germination and dehulling on nutrients, antinutrients, in vitro iron and calcium bioavailability and in vitro starch and protein digestibility of some legume seeds. LWT ‐ Food Science and Technology, 40(7), 1292–1299. 10.1016/j.lwt.2006.08.002

[fsn3856-bib-0022] Hemalatha, S. , Platel, K. , & Srinivasan, K. (2007). Influence of germination and fermentation on bioaccessibility of zinc and iron from food grains. European Journal of Clinical Nutrition, 61, 342–348. 10.1038/sj.ejcn.1602524 16969377

[fsn3856-bib-0023] Hotz, C. , & Gibson, R. S. (2001). Complementary feeding practices and dietary intakes of weanlings in rural Malawi Complementary feeding practices and dietary intakes from complementary foods amongst weanlings in rural Malawi. European Journal Of Clinical Nutrition, 55(10), 841–849. 10.1038/sj.ejcn.1601239 11593345

[fsn3856-bib-0024] Hotz, C. , & Gibson, R. S. (2007). Traditional food‐processing and preparation practices to enhance the bioavailability of micronutrients in plant‐based diets. The Journal of Nutrition, 137(8), 1097–1100. 10.1093/jn/137.4.1097 17374686

[fsn3856-bib-0025] Hotz, C. , Lubowa, A. , Sison, C. , Moursi, M. , & Loechl, C. (2012). A food composition table for Central and Eastern Uganda. Harvest plus technical monograph 9.

[fsn3856-bib-0026] Ikujenlola, A. V. (2014). Chemical and functional properties of complementary food blends from malted and unmalted acha (Digitaria exilis), soybean (Glycine max) and defatted sesame (Sesamun indicum L.) flours. African Journal of Food Science, 8(7), 361–367. 10.5897/ajfs2014.1173 10.5897/ajfs2014.1173

[fsn3856-bib-0027] James, S. , Oloyede, O. O. , Ocheme, O. B. , Chinma, C. E. , & Agbejule, A. Y. (2015). Proximate, anti‐nutrient and sensory properties of ogi, a millet based gruel supplemented with treated African oil bean (Pentaclethra macrophylla Benth.) seed flour. African Journal of Food Science, 9(3), 136–141. 10.5897/ajfs2014.1249 10.5897/ajfs2014.1249

[fsn3856-bib-0028] Kounnavong, S. , Sunahara, T. , Mascie‐Taylor, C. G. N. , Hashizume, M. , Okumura, J. , Moji, K. , & Yamamoto, T. (2011). Effect of daily versus weekly home fortification with multiple micronutrient powder on haemoglobin concentration of young children in a rural area, Lao People's Democratic Republic: A randomised trial. Nutrition Journal, 10(1), 129 10.1186/1475-2891-10-129 22111770 PMC3266642

[fsn3856-bib-0029] Krishnan, R. , Dharmaraj, U. , & Malleshi, N. G. (2012). Influence of decortication, popping and malting on bioaccessibility of calcium, iron and zinc in finger millet. LWT ‐ Food Science and Technology, 48, 169–174. 10.1016/j.lwt.2012.03.003

[fsn3856-bib-0030] Laminu, H. H. , Modu, S. , & Numan, A. I. (2011). Production, in vitro protein digestibility, phytate content and acceptability of weaning foods prepared from pearl millet (Pennisetum typhoideum) and cowpea (Vigna unguiculata). International Journal of Nutrition and Metabolism, 3(October), 109–113.

[fsn3856-bib-0031] Laurie, S. M. , & Van Heerden, S. M. (2012). Consumer acceptability of four products made from beta‐carotene‐rich sweet potato. African Journal of Food Science, 6(4), 96–103. 10.5897/AJFS12.014

[fsn3856-bib-0032] Lim, J. (2011). Hedonic scaling: A review of methods and theory. Food Quality and Preference, 22(8), 733–747. 10.1016/j.foodqual.2011.05.008

[fsn3856-bib-0033] Makanjuola, O. M. , Ogunmodede, A. S. , Makanjuola, J. O. , & Awonorin, S. O. (2012). Comparative study on quality attributes of gari obtained from some processing centres in south west, Nigeria. Advance Journal of Food Science and Technology, 4(3), 135–140.

[fsn3856-bib-0034] Mamiro, P. , Mwanri, A. , Mamiro, D. , Nyagaya, M. , & Ntwenya, J. (2016). In‐vitro bioavailability of selected minerals in dry and green shelled beans. African Journal of Agricultural Research, 11(9), 730–737. 10.5897/AJAR2012.883

[fsn3856-bib-0035] McEwan, R. , Shangase, F. N. , Djarova, T. , & Opoku, A. R. (2014). Effect of three processing methods on some nutrient and anti‐nutritional factor constituent of Colocasia esculenta (Amadumbe). African Journal of Food Science, 8(5), 287–292. 10.5897/AJFS2013.1139

[fsn3856-bib-0036] Mouquet, C. , & Trèche, S. (2001). Viscosity of gruels for infants: A comparison of measurement procedures. International Journal of Food Sciences and Nutrition, 52, 389–400. 10.1080/09637480120078276 11517731

[fsn3856-bib-0037] Mugendi, J. B. , Njagi, E. N. M. , Kuria, E. N. , Mwasaru, M. A. , & Mureithi, J. G. (2010). Effects of processing technique on the nutritional composition and anti‐nutrient content of mucuna bean (Mucuna pruriens L.). African Journal of Food Science, 4(4), 156–166.

[fsn3856-bib-0038] Nkundabombi, M. G. , Nakimbugwe, D. , & Muyonga, J. H. (2015). Effect of processing methods on nutritional, sensory, and physicochemical characteristics of biofortified bean flour. Food Science & Nutrition, 4(3), 384–397. 10.1002/fsn3.301 27247769 PMC4867759

[fsn3856-bib-0039] Olagunju, A. I. , & Ifesan, B. O. T. (2013). Nutritional composition and acceptability of cookies made from wheat flour and germinated sesame (Sesamum indicum) flour blends. British Journal of Applied Science & Technology, 3(34), 702–713. 10.9734/BJAST

[fsn3856-bib-0040] Onweluzo, J. C. , & Nwabugwu, C. C. (2009). Fermentation of millet (Pennisetum americanum) and pigeon pea (Cajanus cajan) seeds for flour production: Effects on composition and selected functional properties. Pakistan Journal of Nutrition, 8(6), 737–744.

[fsn3856-bib-0041] Onyango, C. A. , Ochanda, S. O. , Mwasaru, M. A. , Ochieng, J. K. , Mathooko, F. M. , & Kinyuru, J. N. (2013). Effects of malting and fermentation on anti‐nutrient reduction and protein digestibility of red sorghum, white sorghum and pearl millet. Journal of Food Research, 2(1), 41–49. 10.5539/jfr.v2n1p41

[fsn3856-bib-0042] Polshettiwar, S. A. , Ganjiwale, R. O. , Wadher, S. J. , & Yeole, P. G. (2007). Spectrophotometric estimation of total tannins in some ayurvedic eye drops. Indian Journal of Pharmaceutical Sciences, 69(August), 574–576.

[fsn3856-bib-0043] Saha, K. K. , Frongillo, E. A. , Alam, D. S. , Arifeen, S. E. , Persson, L. Å. , & Rasmussen, K. M. (2008). Appropriate infant feeding practices result in better growth of infants and young children in rural Bangladesh. The American Journal of Clinical Nutrition, 87(12), 1852–1859. 10.1093/ajcn/87.6.1852 18541577 PMC2518656

[fsn3856-bib-0044] Thapliyal, V. , & Singh, K. (2015). Finger millet: Potential millet for food security and power house of nutrients. International Journal of Research in Agriculture and Forestry, 2(2), 22–33.

[fsn3856-bib-0045] Ting, S. R. , & Loh, S. P. (2016). In vitro bioaccessibility of calcium, iron and zinc from breads and bread spreads. International Food Research Journal, 23(5), 2175–2180.

[fsn3856-bib-0046] Tizazu, S. , Urga, K. , Abuye, C. , & Retta, N. (2010). Improvement of energy and nutrient density of sorghum‐based complementary foods using germination. African Journal of Food Agriculture Nutrition and Development, 10(8), 2927–2942.

[fsn3856-bib-0047] Tong, A. , Sainsbury, P. , & Craig, J. (2007). Consolidated criteria for reporting qualitative research (COREQ): A 32‐item checklist for interviews and focus groups. International Journal for Quality in Health Care, 19(6), 349–357. 10.1093/intqhc/mzm042 17872937

[fsn3856-bib-0048] Turyashemererwa, F. M. , Kikafunda, J. K. , & Agaba, F. E. (2009). Prevalence of early childhood malnutrition and influencing factors in Peri urban areas of Kabarole district, Western Uganda. African Journal of Food, Agriculture, Nutrition and Development, 9(4), 975–989.

[fsn3856-bib-0049] Van Eys, J. E. (2005). Manual of quality analyses for soybean products in the feed industry (second). Fourqueux, France: GANS.

[fsn3856-bib-0050] WHO (2009). Infant and young child feeding: Model Chapter for textbooks for medical students and allied health professionals, vol. 155 Geneva, Switzerland: WHO Press. 23905206

